# Branched-chain amino acids prevent hepatic fibrosis and development of hepatocellular carcinoma in a non-alcoholic steatohepatitis mouse model

**DOI:** 10.18632/oncotarget.15304

**Published:** 2017-02-13

**Authors:** Kai Takegoshi, Masao Honda, Hikari Okada, Riuta Takabatake, Naoto Matsuzawa-Nagata, Jean S. Campbell, Masashi Nishikawa, Tetsuro Shimakami, Takayoshi Shirasaki, Yoshio Sakai, Taro Yamashita, Toshinari Takamura, Takuji Tanaka, Shuichi Kaneko

**Affiliations:** ^1^ Department of Gastroenterology, Kanazawa University Graduate School of Medicine, Kanazawa, Japan; ^2^ Department of Advanced Medical Technology, Kanazawa University Graduate School of Health Medicine, Kanazawa, Japan; ^3^ Department of Disease Control and Homeostasis, Kanazawa University Graduate School of Medicine, Kanazawa, Japan; ^4^ Department of Pathology, University of Washington School of Medicine, Seattle, WA, USA; ^5^ The Tohkai Cancer Research and Prevention, Gifu, Japan

**Keywords:** branched-chain amino acids, nonalcoholic steatohepatitis, mammalian target of rapamycin complex 1, hepatocellular carcinoma, transforming growth factor β1

## Abstract

Oral supplementation with branched-chain amino acids (BCAA; leucine, isoleucine, and valine) in patients with liver cirrhosis potentially suppresses the incidence of hepatocellular carcinoma (HCC) and improves event-free survival. However, the detailed mechanisms of BCAA action have not been fully elucidated. BCAA were administered to atherogenic and high-fat (Ath+HF) diet-induced nonalcoholic steatohepatitis (NASH) model mice. Liver histology, tumor incidence, and gene expression profiles were evaluated. Ath+HF diet mice developed hepatic tumors at a high frequency at 68 weeks. BCAA supplementation significantly improved hepatic steatosis, inflammation, fibrosis, and tumors in Ath+HF mice at 68 weeks. GeneChip analysis demonstrated the significant resolution of pro-fibrotic gene expression by BCAA supplementation. The anti-fibrotic effect of BCAA was confirmed further using platelet-derived growth factor C transgenic mice, which develop hepatic fibrosis and tumors. *In vitro*, BCAA restored the transforming growth factor (TGF)-β1-stimulated expression of pro-fibrotic genes in hepatic stellate cells (HSC). In hepatocytes, BCAA restored TGF-β1-induced apoptosis, lipogenesis, and Wnt/β-Catenin signaling, and inhibited the transformation of WB-F344 rat liver epithelial stem-like cells. BCAA repressed the promoter activity of TGFβ1R1 by inhibiting the expression of the transcription factor NFY and histone acetyltransferase p300. Interestingly, the inhibitory effect of BCAA on TGF-β1 signaling was mTORC1 activity-dependent, suggesting the presence of negative feedback regulation from mTORC1 to TGF-β1 signaling. Thus, BCAA induce an anti-fibrotic effect in HSC, prevent apoptosis in hepatocytes, and decrease the incidence of HCC; therefore, BCAA supplementation would be beneficial for patients with advanced liver fibrosis with a high risk of HCC.

## INTRODUCTION

The recent increase in non-alcoholic fatty liver disease (NAFLD) associated with metabolic syndrome could represent a high risk factor for HCC [1]. The development of hepatic steatosis associated with inflammatory changes, called non-alcoholic steatohepatitis (NASH), can lead to liver cirrhosis and hepatocellular carcinoma (HCC). However, the pathogenesis of NASH is still unclear and an effective treatment for NASH has not been established.

The lack of an appropriate experimental model of HCC that develops on a background of NASH could be a barrier to the development of effective therapies for preventing and treating NASH-derived HCC. To address these limitations, we previously reported an atherogenic combined with high-fat (Ath+HF) diet mouse model that develops hepatic steatosis, inflammation, fibrosis, and insulin resistance [2]. The liver histology of Ath+HF diet mice showed cellular ballooning, a necessary histological feature defining human NASH [2]. Therefore, the Ath+HF diet mouse model resembles human NASH and has the potential to be used for the study of NASH-derived HCC.

Long-term supplementation with branched-chain amino acids (BCAA; leucine, isoleucine, and valine) in patients with liver cirrhosis is reported to improve their nutritional status as well as event-free survival [3]. A recent report revealed that BCAA suppressed the incidence of HCC in obese patients with hepatitis C virus-related cirrhosis [4]. In rodent models, BCAA suppressed diethylnitrosamine (DEN)-induced liver tumorigenesis in mouse [5, 6] and rat [7]. Although studies showing the suppressive effect of BCAA on the development of HCC are increasing, the detailed mechanisms by which BCAA suppress HCC have not been fully addressed. In this study, we used the Ath+HF diet-induced NASH-HCC mouse model to investigate the effect of BCAA on these mice. We found that BCAA inhibited pro-fibrotic signaling and tumorigenesis by inhibiting transforming growth factor (TGF)-β1 signaling.

## RESULTS

### BCAA supplementation improved steatosis, inflammation, and fibrosis in the liver of the Ath+HF diet mouse model

**Figure 1:**
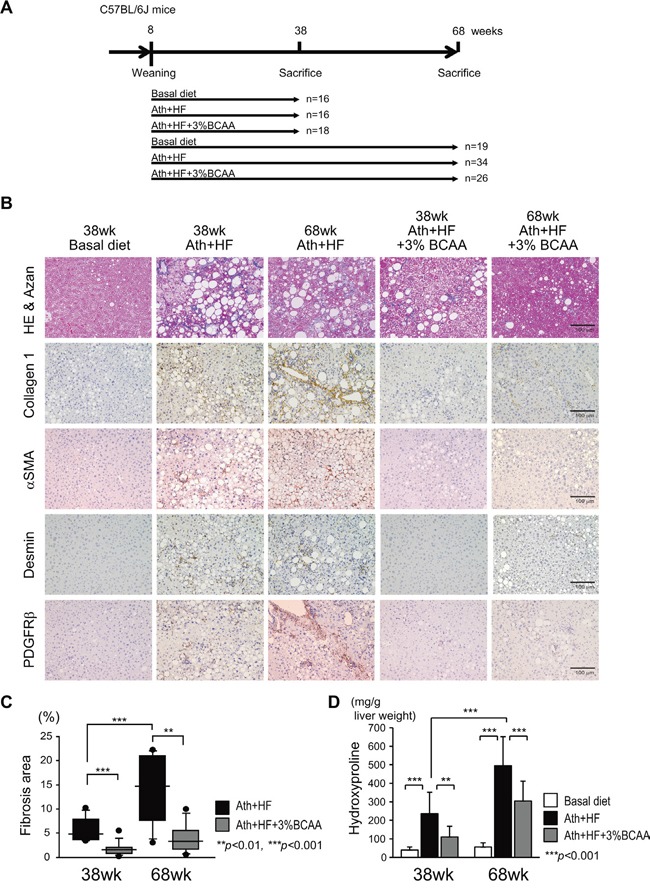
Histological improvement in the liver of Ath+HF diet mice by BCAA supplementation **A**. Feeding schedule of the mice. After weaning, male C57BL/6J mice were divided randomly into 3 groups: (i) basal diet, (ii) Ath+HF diet, and (iii) Ath+HF diet supplemented with 3% BCAA. **B**. Azan staining of mouse livers, IHC staining for collagen 1, α-SMA, desmin, and PDGFRβ expression in livers of mice fed the basal, Ath+HF, or Ath+HF diet supplemented with 3% BCAA at 38w and 68w. **C**. Densitometric analysis of liver fibrotic areas of mice fed the Ath+HF diet or Ath+HF diet supplemented with 3% BCAA at 38w and 68 w (N = 8). **D**. Hydroxyproline content of mice liver fed the Ath+HF diet or Ath+HF diet supplemented with 3% BCAA at 38w and 68 w (N = 8).

**Figure 2:**
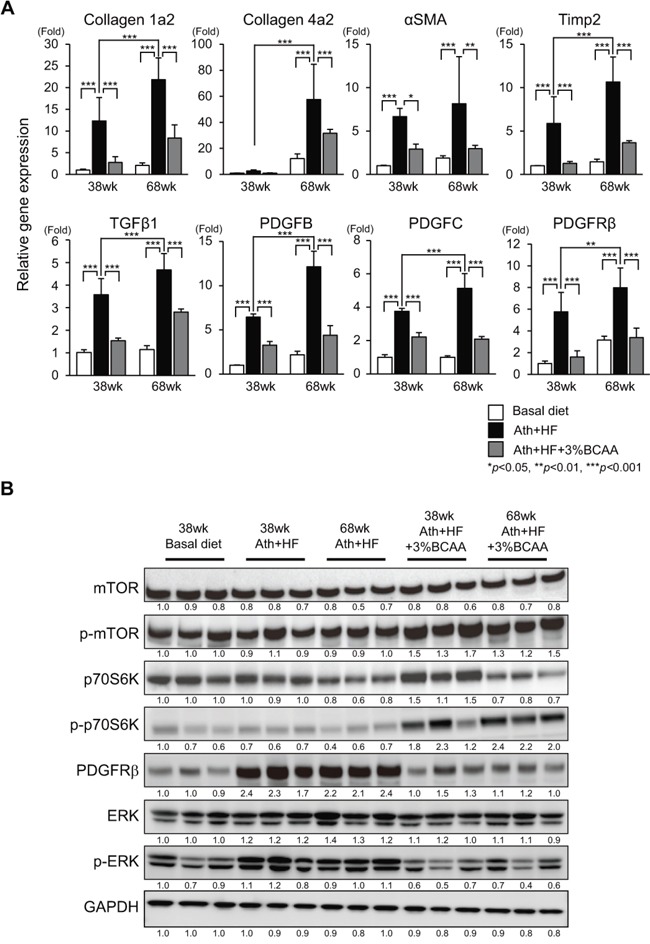
Effects of BCAA supplementation on liver fibrosis, mTORC1, and PDGFRβ signaling in C57BL/6J mice fed the Ath+HF diet **A**. Relative expression of mRNA for collagen 1a2, collagen 4a2, α-SMA, tissue inhibitor of metalloproteinase 2, TGF-β1, PDGFB, PDGFC, and PDGFRβ in livers of mice fed the basal, Ath+HF, or Ath+HF diet supplemented with 3% BCAA at 38w and 68w (N = 5). **B**. Western blotting of mTOR, p-mTOR, p70S6K, p-p70S6K, PDGFRβ, ERK, and p-ERK in livers of mice fed the basal, Ath+HF, or Ath+HF diet supplemented with 3% BCAA at 38w and 68w.

Male C57BL/6J mice after weaning at week 8 were fed a basal diet (basal diet group), Ath+HF diet (Ath+HF group), or Ath+HF diet containing 3% BCAA (Ath+HF+BCAA group), respectively (Figure 1A). The mice were killed at 38w or 68w for the evaluation of liver histology or tumor incidence. Hematoxylin and eosin and Azan staining of formalin-fixed paraffin-embedded liver sections showed that substantial steatosis and fibrosis were observed in the liver of the Ath+HF group and were less severe in the Ath+HF+BCAA group (Figure 1B). The area of fibrosis deduced from densitometry analysis and the hydroxyproline content in the liver revealed the progression of hepatic fibrosis from 38w to 68w, and hepatic fibrosis was significantly reduced in the Ath+HF+BCAA group compared with the Ath+HF group (Figure 1C and 1D). Laboratory data for the serum of 12w and 68w mice (Supplementary Table 1) showed that the levels of serum alanine aminotransferase (ALT), plasma total cholesterol, and free cholesterol were significantly up-regulated in the Ath+HF group compared with the basal diet group, and the values were significantly reduced in the Ath+HF+BCAA group in 68w mice. Immunohistochemical (IHC) staining of collagen 1, alpha smooth muscle actin (α-SMA), desmin, and platelet-derived growth factor receptor (PDGFR) β in the liver showed that these fibrosis markers were substantially up-regulated in the liver of the Ath+HF group and repressed in the Ath+HF+BCAA group (Figure 1B). Quantitative RT-PCR (qRT-PCR) analysis showed that the expression of collagen 1a2, collagen 4a2, α-SMA, tissue inhibitor of metalloproteinase 2, TGF-β1, PDGFB, PDGFC, and PDGFRβ mRNA was significantly up-regulated in the Ath+HF group and their expression was repressed in the Ath+HF+BCAA group (Figure 2A). Western blotting analysis showed the up-regulation of the phosphorylated forms of mTOR (p-mTOR) and ribosomal protein S6 kinase (p-p70S6K) in the Ath+HF+BCAA group, confirming the activation of mammalian target of rapamycin complex 1 (mTORC1) signaling by BCAA supplementation (Figure 2B). Conversely, the expression of PDGFRβ and the phosphorylated form of extracellular signal-regulated kinase (p-ERK) was down-regulated in the Ath+HF+BCAA group compared with the Ath+HF group.

The global changes of gene expression in the liver of the Ath+HF and Ath+HF+BCAA groups were analyzed using an Affymetrix GeneChip (Supplementary Figure 1). Gene expression profiling in the liver of the Ath+HF group at 68w was compared with that of the basal diet group. We found that 1409 genes were up-regulated and 355 genes were down-regulated in the Ath+HF group (fold > 1.5, P < 0.005). One-way hierarchical clustering of the differentially expressed genes showed the dynamic change of gene expression associated with the progression of liver disease (from 38w to 68w). The expression of the up-regulated genes, including many inflammation- and fibrosis-related genes, increased with the progression of liver disease, and BCAA supplementation repressed this up-regulation. Pathway analysis of the up-regulated gene clusters using MetaCore showed that the pathways related to epithelial-mesenchymal transition, extracellular matrix remodeling, chemokines and cell adhesion, and phosphatidylinositol diphosphate pathway were up-regulated (Supplementary Table 2). In addition to pro-fibrotic genes, oncogenes and metastasis-related genes such as p21 protein activated kinase 1, vimentin, phosphoinositide-3-kinase, Vav1 oncogene, and thymoma viral proto oncogene (Akt), were up-regulated (Supplementary Figure 1). Conversely, the expression of the down-regulated genes, including many metabolism- (e.g., leptin receptor and insulin receptor substrate 2) and mitochondria-related genes, decreased with the progression of liver disease, and BCAA supplementation rescued this down-regulation (Supplementary Figure 1 and Supplementary Table 2). These results demonstrated that long-term dietary exposure to the Ath+HF diet induced the progression of fibrosis and precancerous oncogenic signaling accompanied with metabolism-related gene abnormalities. BCAA supplementation restored the expression of these genes.

### BCAA supplementation prevented the occurrence of liver tumors in the Ath+HF diet mouse model

**Figure 3:**
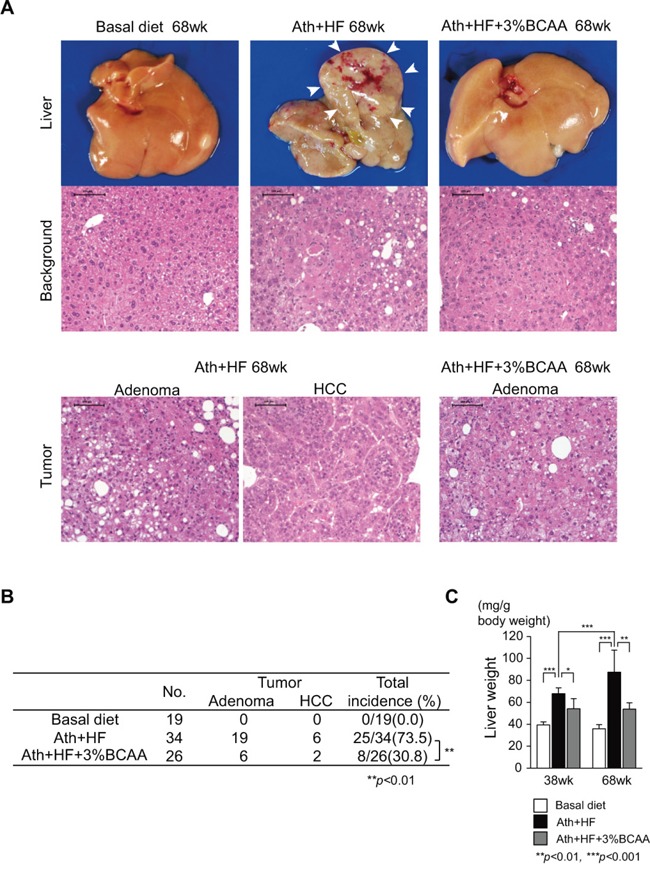
Effects of BCAA supplementation on liver tumorigenesis in C57BL/6J mice fed the Ath+HF diet **A**. Macroscopic findings of livers and hematoxylin and eosin staining of background livers and hepatic tumors. **B**. Incidence of hepatic tumors (adenoma or HCC) in livers of mice that were fed the basal, Ath+HF, or Ath+HF diet supplemented with 3% BCAA at 68w. **C**. Liver weights of mice fed the basal, Ath+HF, or Ath+HF diet supplemented with 3% BCAA at 38w and 68w.

We evaluated liver tumors in the basal, Ath+HF, and Ath+HF+BCAA diet groups at 68w (Figure 3). The appearance of the liver surface in the Ath+HF group was nodular and HCC was observed in the liver (Figure 3A). BCAA supplementation improved the appearance of the liver. Histological assessment of liver tumors revealed the typical morphology of hepatic adenoma or HCC (Figure 3A). The Ath+HF group developed liver tumors at a high frequency (73.5%), while no liver tumors were observed in the basal diet group. BCAA supplementation significantly reduced the incidence of liver tumors (30.8%, P < 0.01) (Figure 3B). In addition, BCAA supplementation significantly reduced the increase of liver weight in the Ath+HF group (Figure 3C).

### BCAA supplementation reduced the incidence of fibrosis and tumors in the liver of *Pdgf-c Tg* mice

To examine the effect of BCAA on a different NASH-HCC mouse model, we utilized *Pdgf-c Tg* mice, which develop hepatic fibrosis, steatosis, and tumors [8]. *WT* and *Pdgf-c Tg* mice were fed a basal diet (*WT* basal diet group and *Pdgf-c Tg* basal diet group, respectively) or a basal diet supplemented with 3% BCAA (*WT* BCAA group and *Pdgf-c Tg* BCAA group, respectively) (Supplementary Materials and Methods) (Supplementary Figure 2A). Hepatic fibrosis and tumor incidence were evaluated at 28w. The *Pdgf-c Tg* basal diet group developed hepatic fibrosis, whereas no fibrosis was observed in the *WT* basal diet group. The area of fibrosis was significantly reduced in the *Pdgf-c Tg* BCAA group compared with the *Pdgf-c Tg* basal diet group, while serum ALT levels were not different between the two groups (Supplementary Table 3, Figure 2B and 2C). The expression of collagen 1a2, collagen 4a2, α-SMA, and PDGFRβ mRNA was significantly up-regulated in the *Pdgf-c Tg* basal diet group compared with the *WT* basal diet group, and this up-regulation was significantly reduced in the *Pdgf-c Tg* BCAA group (Supplementary Figure 2D). Western blotting analysis showed the up-regulated expression of PDGFRβ, p300, p-ERK, and α-SMA in the *Pdgf-c Tg* basal diet group compared with the *WT* basal diet group, and this up-regulation was reduced in the *Pdgf-c Tg* BCAA group (Supplementary Figure 2E). Similarly, IHC staining showed the up-regulation of collagen 1, desmin, and PDGFRβ in the *Pdgf-c Tg* basal diet group, and this up-regulation was repressed in the *Pdgf-c Tg* BCAA group (Supplementary Figure 3).

The appearance of the liver in the *Pdgf-c Tg* basal diet group was associated with multiple nodules, while it was much improved in the *Pdgf-c Tg* BCAA group (Supplementary Figure 4A). Actually, the *Pdgf-c Tg* basal diet group developed hepatic tumors at 100% (8 of adenoma and 1 of HCC), while the *Pdgf-c Tg* BCAA group developed only 1 tumor (11.1%) (Supplementary Figure 4B). Liver weight increased in the *Pdgf-c Tg* basal diet group, and this increase was significantly reduced in the *Pdgf-c Tg* BCAA group (Supplementary Figure 4C).

### BCAA diminished the pro-fibrotic signaling induced by TGF-β1 in HSC

**Figure 4:**
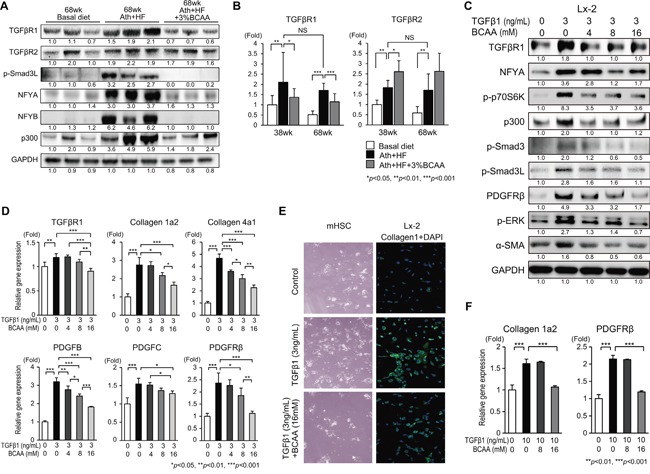
Effects of BCAA on TGF-β1-related signaling in Ath+HF diet mice (A, B), Lx-2 cells (C–E), and primary mouse HSC (E, F) **A**. Western blotting of TGFβR1, TGFβR2, p-Smad3L, NFYA, NFYB, and p300 in livers of mice fed the basal, Ath+HF, or Ath+HF diet supplemented with 3% BCAA at 68w. **B**. Relative expression of mRNA for TGFβR1 and TGFβR2 in livers of mice fed the basal, Ath+HF, or Ath+HF diet supplemented with 3% BCAA at 38w and 68w (N = 8). **C**. Western blotting of TGFβR1, p-p70S6K, p300, p-Smad3, p-Smad3L, PDGFRβ, p-ERK, and α-SMA in Lx-2 cells activated by recombinant human TGF-β1 (3 ng/mL) with different concentrations of BCAA (0, 4, 8 and 16 mM). **D**. Relative expression of mRNA for TGFβR1, collagen 1a2, collagen 4a1, PDGFB, PDGFC, PDGFRβ, and TGFβR2 in Lx-2 cells activated by recombinant human TGF-β1 (3 ng/mL) with different concentrations of BCAA (0, 4, 8, and 16 mM) (N = 3). **E**. Microscopic view of primary mouse HSC treated with recombinant mouse TGF-β1 (3 ng/mL) with or without BCAA (16 mM) for 24 h (left). IF staining for collagen 1 in Lx-2 cells activated by recombinant human TGF-β1 (3 ng/mL) with or without BCAA (16 mM) for 24 h (right). **F**. Relative expression of mRNA for collagen 1a2 and PDGFRβ in primary mouse HSC treated with recombinant mouse TGFβ with or without BCAA (N = 3).

To examine the mechanism of the anti-fibrotic and anti-tumor effects of BCAA on HCC development, we focused on genes with a shared function of pro-fibrotic, metabolic, and oncogenic signaling. Genes related to TGF-β1 signaling, such as TGFβR1, TGFβR2, and p-Smad3L, genes related to TGF-β1 and metabolism-related transcription factors (NFYA and NFYB), and a gene related to TGF-β1 and WNT/β-catenin-related histone acetyltransferase (p300) were evaluated (Figure 4A). The expression of these genes, except TGFβR2, was up-regulated in the Ath+HF group and repressed in the Ath+HF+BCAA group (Figure 4A). qRT-PCR analysis of TGFβR1 and TGFβR2 showed the significant up-regulation of TGFβR1 in the Ath+HF group, and its expression was significantly repressed in the Ath+HF+BCAA group at both 38w and 68w (Figure 4B), while no significant reduction of TGFβR2 was observed in the Ath+HF+BCAA group.

The influence of BCAA on TGF-β1 signaling was evaluated in the human HSC cell line Lx-2 (Figure 4C–4E). TGF-β1 increased the expression of TGFβR1, p-p70S6K, p300, p-Smad3, p-Smad3L, PDGFRβ, p-ERK, and αSMA in Lx-2 cells, and this activation was repressed by the addition of BCAA (Figure 4C). Correlating with these results, the expression of collagen 1a2, collagen 4a1, PDGFB, and PDGFC was up-regulated by TGF-β1, and this up-regulation was repressed by the addition of BCAA in Lx-2 cells, as demonstrated by qRT-PCR analysis (Figure 4D) and immunofluorescent (IF) staining (Figure 4E). The addition of BCAA repressed the TGF-β1-induced trans-differentiation of HSC to myofibroblast-like cells (Figure 4E). An MTT assay showed that TGF-β1 increased the cell viability of primary mouse HSC and Lx-2 cells, while it decreased the viability of primary mouse hepatocytes. The addition of BCAA restored the changes of cell viability induced by TGF-β1 (Supplementary Figure 5). The expression of collagen 1a2 and PDGFRβ was up-regulated by TGF-β1 in primary mouse HSC, and this up-regulation was repressed by the addition of BCAA (Figure 4F). These results showed that BCAA attenuated TGF-β1-stimulated signaling in HSC.

### BCAA diminished the lipogenesis, WNT/β-catenin, and pro-apoptotic signaling induced by TGF-β1 in hepatocytes

**Figure 5:**
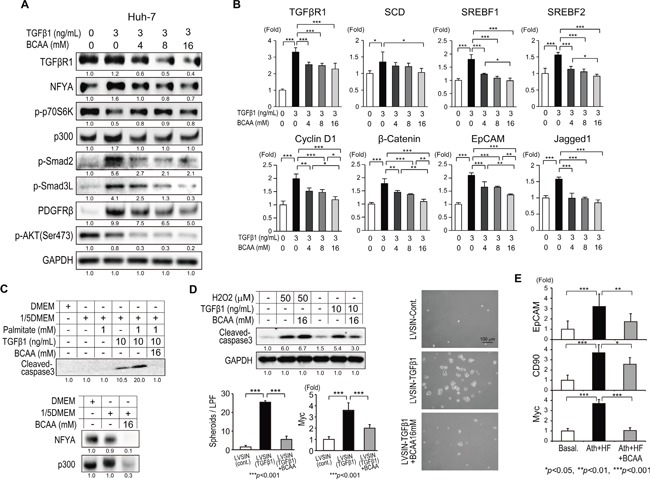
Effects of BCAA on TGF-β1-related signaling in hepatocytes **A**. Western blotting of TGFβR1, p-p70S6K, p300, p-Smad2, p-Smad3L, PDGFRβ, and p-AKT(Ser473) in Huh-7 cells activated by recombinant human TGF-β1 (3 ng/mL) with different concentrations of BCAA (0, 4, 8, and 16 mM). **B**. Relative expression of mRNA for TGFβR1, SCD, SREBF1, SREBF2, cyclin D1, β-catenin, EpCAM, and Jagged 1 in Lx-2 cells activated by recombinant human TGF-β1 (3 ng/mL) with different concentrations of BCAA (0, 4, 8, and 16 mM) (N = 3). **C**. Western blotting of cleaved caspase-3 (upper), NFYA, and p300 (lower) in primary mouse hepatocytes under stimulation with 1/5 DMEM, palmitate, and recombinant mouse TGF-β1 with or without BCAA. **D**. Western blotting of cleaved caspase-3 in WB-F344 cells under stimulation with H_2_O_2_ or recombinant mouse TGF-β1 with or without BCAA (left-upper). Representative view of spheroid formation of WB-F344 cells transduced by recombinant lentivirus (LVSIN-TGF-β1) with or without BCAA (right). Number of spheroids formed by WB-F344 cells and Myc expression in spheroids (N = 5) (left-lower). **E**. Expression of the tumor-initiating markers EpCAM, CD90, and Myc in livers of mice fed the basal, Ath+HF, or Ath+HF diet supplemented with 3% BCAA at 68w (N = 10).

The influence of BCAA on TGF-β1 signaling was evaluated in the human hepatocellular carcinoma Huh-7 cell line (Figure 5A and 5B). BCAA attenuated TGF-β1 signaling (TGFβRI, NFYA, p300, p-Smad2, p-Smad3L, PDGFRβ, and p-AKT) in Huh-7 cells. In contrast to the results from Lx-2 cells (Figure 4C), TGF-β1 repressed the expression of p-p70S6K, and BCAA partially restored its expression (Figure 5A). Interestingly, TGF-β1 stimulated the expression of lipogenesis transcription factors such as SCD, SREBF1, and SREBF2 (Figure 5B). In addition, TGF-β1 stimulated the expression of WNT/β-catenin signaling-related genes such as cyclin D1, β-catenin, EpCAM, and Jagged 1 in Huh-7 cells (Figure 5B). BCAA reduced the expression of these genes significantly (Figure 5B). In mouse primary hepatocytes, TGF-β1 increased the expression of cleaved caspase 3 in the amino acid-depleted condition (1/5 DMEM) (Figure 5C upper). Palmitate accelerated the expression of cleaved caspase 3 induced by TGF-β1, while BCAA completely repressed this expression (Figure 5C upper). Importantly, BCAA substantially repressed the expression of NFYA and p300 in mouse primary hepatocytes (Figure 5C lower). Thus, BCAA repressed TGF-β1-induced lipogenesis, WNT/β-catenin, and pro-apoptotic signaling in hepatocytes.

To examine the role of BCAA in hepatocarcinogenesis, we utilized WB-F344 rat liver epithelial stem-like cells [9]. Hydrogen peroxide (H_2_O_2_) treatment induced the expression of cleaved caspase 3 in WB-F344 cells; however, it was not repressed by the addition of BCAA. Conversely, BCAA repressed the TGF-β1-induced expression of cleaved caspase 3 (Figure 5D upper-left). We generated a recombinant lentivirus expressing mouse TGF-β1 (LVSIN-TGF-β1). WB-F344 cells transduced with TGF-β1 formed an increased number of spheroids compared to WB-F344 cells transduced with the control virus (LVSIN-cont). The expression of Myc was significantly increased in TGF-β1-transduced spheroids. The addition of BCAA significantly decreased the number of spheroids and the expression of Myc (Figure 5D lower-left and right). Correlating with these results *in vitro*, the expression of the tumor-initiating markers EpCAM, CD90, and Myc was significantly increased in the Ath+HF group compared with the basal diet group and their expression was significantly repressed in the Ath+HF+BCAA group (Figure 5E).

### BCAA targets TGFβRI by inhibiting NFY and p300

**Figure 6:**
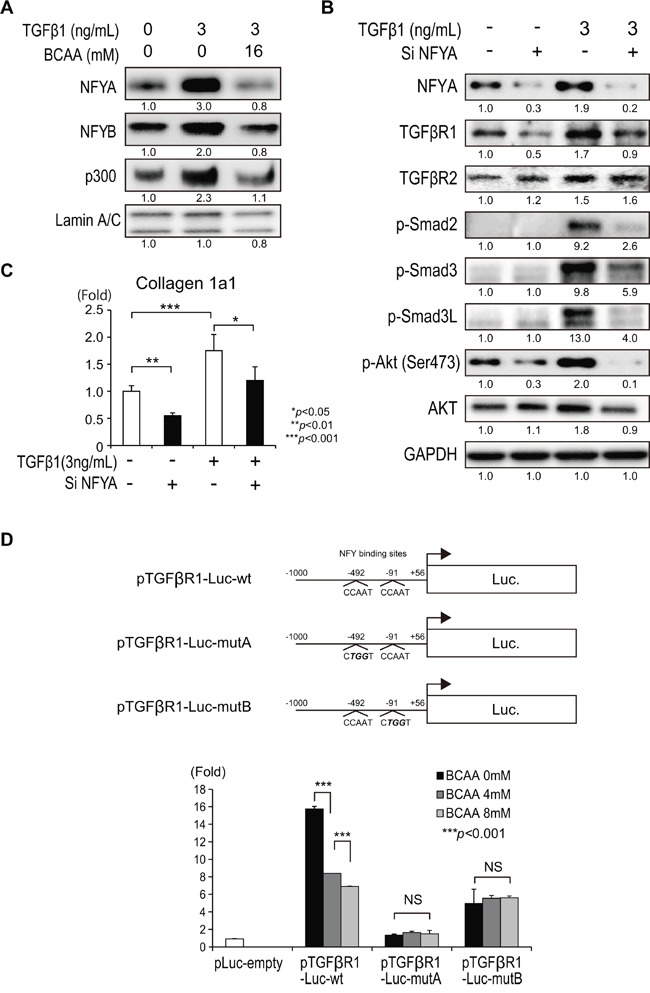
Regulation of TGF-β1 signaling by the transcription factor NFY **A**. Western blotting of NFYA, NFYB, and p300 in the nuclear fraction of Lx-2 cells activated by recombinant human TGF-β1 (3 ng/mL) with or without BCAA (16 mM). **B**. Western blotting of TGFβR1, TGFβR2, p-Smad2, p-Smad3, p-Smad3L, p-AKT(Ser473), and AKT in Lx-2 cells with or without recombinant human TGF-β1 and/or small interfering (si) RNA against NFYA (si NFYA). **C**. Relative expression of mRNA for collagen 1a in Lx-2 cells with or without recombinant human TGF-β1 and/or si NFYA. (N = 3). **D**. Construction of TGFβR1 promoter assay constructs. pTGFβR1-Luc-wt: including −1000 to 56 bp relative to the transcription initiation site of TGFβR1 fused to a firefly luciferase gene. pTGFβR1-Luc-mutA: having mutations at the putative NFY binding site at −492 in pTGFβR1-Luc-wt. pTGFβR1-Luc-mutB: having mutations at the putative NFY binding site at −91 in pTGFβR1-Luc-wt (upper). Dose-dependent inhibition of TGFβR1 promoter activity by BCAA (0, 4, and 8 mM) and the loss of the regulation by BCAA in pTGFβR1-Luc-mutA and pTGFβR1-Luc-mutB (N = 8) (lower).

NFY binds to CCAAT motifs in the promoter region of a variety of genes. NFY is a heteromeric protein composed of three subunits, NFYA, NFYB, and NFYC, which are all necessary for CCAAT binding [10]. Among these subunits, NFYA is a regulatory subunit that is acetylated by the histone acetyltransferase p300 [10]. We examined the relationship between the expression of NFYA and TGF-β signaling. TGF-β1 increased the expression of nuclear NFYA, NFYB, and p300 in Lx-2 cells that was repressed by the addition of BCAA (Figure 6A). To examine the functional relevance of NFYA on TGF-β1 signaling in Lx-2 cells, we knocked down the expression of NFYA by small interfering (si) RNA. TGF-β1 increased the expression of NFYA, TGFβR1, TGFβR2, p-Smad2, p-Smad3, p-Smad3L, and p-Akt (Ser473), and knocking down NFYA decreased the expression of these genes, except for TGFβR2 (Figure 6B). The expression of collagen 1a1 was decreased by the repression of NFYA (Figure 6C). These results could indicate that NFYA mediated TGF-β1 signaling by regulating TGFβR1, but not TGFβR2, in Lx-2 cells. To examine the relationship between NFYA and TGFβR1, we performed a promoter assay of TGFβR1. We found two NFY binding sites upstream of the transcription initiation site of TGFβR1 (−492 and −91). A reporter construct including the putative promoter region (−1000 to 56 bp relative to the transcription initiation site of TGFβR1) fused to firefly luciferase (pTGFβR1-Luc-wt) was used to examine the promoter activity of TGFβR1 (Figure 6D). Interestingly, the addition of BCAA reduced the promoter activity of TGFβR1 significantly. Mutation of either the NFY binding site at −492 (pTGFβR1-Luc-mutA) or −91 (pTGFβR1-Luc-mutB) repressed the basal promoter activity of TGFβR1 and inhibited the suppressive effect of BCAA. Thus, BCAA targets TGFβRI by inhibiting NFY and p300.

### Inhibition of TGF-β1 signaling by BCAA is mTORC1-dependent

**Figure 7:**
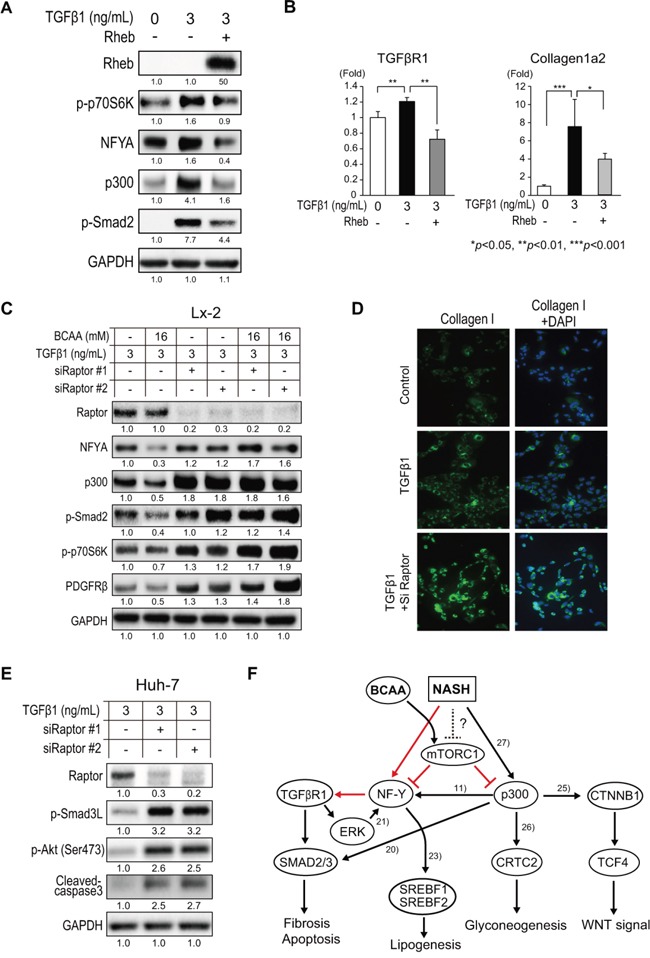
Repression of TGF-β1 signaling by BCAA and mTORC1 activity **A**. Western blotting of Rheb, p-p70S6K, NFYA, p300, and p-Smad2 in Lx-2 cells activated by recombinant human TGF-β1 with or without Rheb overexpression. **B**. Relative expression of mRNA for TGFβR1 and collagen 1a2 in Lx-2 cells activated by human TGF-β1 with or without Rheb overexpression (N = 3). **C**. Western blotting of Raptor, NFYA, p300, p-Smad2, p-p70S6K, and PDGFRβ in TGF-β1-treated Lx-2 cells in which Raptor was knocked down by two siRNAs (siRaptor#1 and siRaptor#2) with or without BCAA. **D**. IF staining of collagen 1a in TGF-β1-treated Lx-2 cells in which Raptor was knocked by siRNA. **E**. Western blotting of Raptor, p-Smad3L, and p-Akt(Ser473) in TGF-β1-treated Lx-2 cells in which Raptor was knocked down by two siRNAs (siRaptor#1 and siRaptor#2). **F**. Putative signaling pathway between BCAA, mTORC1, TGF-β, and WNT signaling in NASH liver. Red lines indicate the regulations that were newly found in this study. Black lines indicate the regulations reported previously.

It is well known that BCAA immediately phosphorylates mTOR and activates mTORC1 signaling [11]; therefore, we examined whether the suppression of TGF-β1 signaling by BCAA was mTORC1-dependent. TGF-β1 increased the expression of p-p70S6K, NFYA, p300, pSmad2 (Figure 7A), TGFβR1, and collagen 1a2 (Figure 7B) in Lx-2 cells. Interestingly, the over-expression of Rheb, an activator of mTORC1, decreased the expression of these genes (Figure 7A and 7B), suggesting that the activation of mTORC1 inhibited TGF-β1 signaling. We next repressed the expression of Raptor, an active component of mTORC1, by using two specific siRNA (siRaptor#1 and siRaptor#2). Both siRNA effectively repressed the expression of Raptor, and, interestingly, TGF-β1 signaling (NFYA, p300, p-Smad2, p-p70S6K, and PDGFRβ) was more activated in cells in which Raptor was knocked down (Figure 7C). The addition of BCAA (16 mM) reduced the expression of TGF-β1-stimulated genes when Raptor was expressed normally; however, BCAA had no additional effect in cells in which Raptor was knocked down (Figure 7C). IF staining showed the increased expression of collagen I in TGF-β1-stimulated cells, and the expression of collagen I was further increased in cells in which was Raptor knocked down (Figure 7D). Similarly, in Huh-7 cells, the expression of p-Smad3L, p-Akt(Ser473), and cleaved-caspase 3 was further increased in Raptor-knocked down cells (Figure 7E). Therefore, inhibition of TGF-β1 signaling by BCAA is mTORC1-dependent.

TGF-β1 increased the expression of p-p70S6K in Lx-2 cells but decreased it in Huh-7 cells (Figure 4C and 5A). Thus, differential mTORC1 signaling was induced by TGF-β1 in HSC and hepatocytes. To explore these findings in more detail, the time course of mTORC1 signaling induced by TGF-β1 was evaluated in Lx-2 and Huh-7 cells (Supplementary Figure 6A). TGF-β1 decreased the expression of p-mTOR and p-p70S6K over 24 h in Huh-7 cells, while their expression was rather maintained in Lx-2 cells (Supplementary Figure 6A). These data indicated that TGF-β1 had an anti-proliferative effect on hepatocytes, while it trans-differentiated HSC into more replicative myofibroblast-like cells, resulting in the increase of mTORC1 signaling. Interestingly, the addition of BCAA diminished the activation of p-Smad2/3 induced by TGF-β1 in both Huh-7 and Lx-2 cells, and suppressed the trans-differentiation of Lx-2 cells to myofibroblast-like cells (Supplementary Figure 6A). These results collectively indicated the presence of negative feedback regulation from mTORC1 to TGF-β1 signaling in HSC, and the regulation of mTORC1 and TGF-β1 signaling would be different between HSC and hepatocytes. In HSC, TGF-β1 signaling activated mTORC1 signaling, while it inhibited mTORC1 signaling in hepatocytes, and mTORC1 inhibited TGF-β1 signaling in both HSC and hepatocytes (Supplementary Figure 6B).

## DISCUSSION

NAFLD and NASH are now the leading causes of chronic liver disease in the West and their prevalence is increasing worldwide, including Japan [1]. NASH can develop into liver cirrhosis and HCC; however, an effective treatment to prevent the progression of NASH has not been established.

It has been accepted that long-term treatment with BCAA is an effective preventive treatment for improving the clinical outcome of cirrhotic patients by reducing the likelihood of liver failure [3, 12, 13]. Moreover, recent reports described that BCAA suppressed the incidence of HCC in cirrhotic patients with or without obesity and insulin resistance [4, 14, 15]. Supporting these findings, several mouse experiments demonstrated that BCAA reduced the incidence of DEN-induced HCC by improving insulin resistance [5], oxidative stress [16], and angiogenesis [7]. However, the precise mechanisms underlying the anti-tumor effect of BCAA have not been elucidated. As human HCC frequently originates from a background liver associated with persistent inflammation and advanced fibrosis, a mouse model resembling human NASH would be ideal for the evaluation of the anti-HCC effect of BCAA. In this study, we established a diet-induced NASH mouse model that exhibits steatosis, inflammation, progression of fibrosis, and eventually hepatic tumors including HCC. Using this model, we examined the effect of BCAA on the progression of NASH.

In this study, we demonstrated the anti-fibrotic and anti-tumor effects of BCAA in two independent mouse models. Our findings will be useful for the development of new therapeutic strategies to prevent the progression of NASH. Although short-term BCAA supplementation (12w) did not improve serum ALT levels, long-term BCAA supplementation (68w) significantly improved hepatic steatosis, inflammation, and fibrosis, and the findings were comparable with the changes of gene expression (Supplementary Figure 1). We confirmed the significant reduction of pro-fibrotic gene expression in Ath+HF diet mice supplemented with BCAA by qRT-PCR, IHC, and western blotting (Figures 1 and 2). Moreover, at 68w, BCAA supplementation significantly reduced the incidence of hepatic tumors from 73.5% to 30.8% (*P* < 0.01) (Figure 3).

One of the characteristic and unique findings of our study was the improvement of hepatic fibrosis by BCAA supplementation. To assess this effect further, we utilized another mouse model, *Pdgf-c Tg*, in which PDGF-C was over-expressed in the liver. *Pdgf-c Tg* mice develop hepatic fibrosis, steatosis, and HCC [8]. BCAA supplementation significantly reduced hepatic fibrosis in the liver of *Pdgf-c Tg* mice. These findings were confirmed by the reduced expression of pro-fibrotic genes using qRT-PCR, IHC, and western blotting (Supplementary Figure 2 and Supplementary Figure 3). Unexpectedly, serum ALT levels were not improved by BCAA supplementation; therefore, the protection of hepatocytes by BCAA might not be a major mechanism for the resolution of hepatic fibrosis. Moreover, BCAA supplementation significantly reduced the incidence of hepatic tumors in *Pdgf-c Tg* mice (Supplementary Figure 4). Recent reports showed that PDGF-C activates TGF-β/Smad3 signaling pathways to regulate HSC proliferation, collagen production, and ultimately fibrosis [17]. TGF-β1 activates PDGF signaling [18]; therefore, these two signaling pathways cooperatively contribute to hepatic fibrosis. Our present results demonstrated that BCAA directly inhibited the fibrosis signaling pathway.

To reveal the molecular target of BCAA, we focused on TGF-β signaling. Genes related to TGF-β1 signaling, such as TGFβR1, p-Smad3L, PDGFRβ, p-ERK [19], NFYA, NFYB, and p300 [20], were up-regulated in the Ath+HF group and repressed in the Ath+HF+BCAA group (Figure 2B and 4A). *In vitro*, BCAA suppressed TGF-β1 signaling in HSC (Lx-2 cells and mouse primary HSC) (Figure 4) as well as in hepatocytes (Huh-7 cells and mouse primary hepatocytes) (Figure 5). The expression of TGF-β1 signaling, PDGF signaling, collagen 1a2, and collagen 4a1 was evaluated quantitatively by qRT-PCR, western blotting, and IHC (Figures 4 and 5).

As for the regulatory molecules of TGF-β1 signaling, we investigated the expression of the transcription factor NFY and histone acetyltransferase p300. We found that BCAA reduced the expression of NFYA and p300 in two mouse NASH models (Figure 4 and Supplementary Figure 2), in two cell lines (Lx-2 and Huh-7 cells, Figures 4C and 5A), and mouse primary hepatocytes (Figure 5C). Although further studies should be performed to clarify the detailed mechanisms by which BCAA inhibited NFYA and p300, we showed the possible interaction of these transcriptional regulators and mTORC1 signaling for the first time.

NFY is a heteromeric protein composed of three subunits (NFYA, NFYB, and NFYC), which are all necessary for CCAAT binding [10]. Among these subunits, NFYA is a regulatory subunit that is acetylated by the histone acetyltransferase p300 [10] (Figure 7F). We found a significant up-regulation of NFYA in liver tissue of NAFLD patients compared with tissue of normal liver patients (data not shown). NFY was reported to regulate TGFβR2 [21]; however, in the present study, BCAA supplementation did not suppress TGFβR2 expression in the Ath+HF+BCAA group (Figure 4A and 4B). Moreover, repression of NFYA expression in Lx-2 cells by siRNA suppressed the expression of TGF-β1 signaling (such as TGFβR1, p-Smad2, p-Smad3L, and p-Akt[Ser473]), except for TGFβR2 (Figure 6B), indicating that NFY may not regulate TGFβR2 in HSC. In contrast, BCAA suppressed the expression of TGFβR1 in the Ath+HF+BCAA group, Lx-2 cells, and Huh-7 cells. Interestingly, we showed that BCAA repressed the promoter activity of TGFβR1, and mutation of the NFY binding sites (−492 and −91) in this promoter abolished the suppressive effect of BCAA on the promoter activity of TGFβR1 (Figure 6D). Therefore, our results showed that BCAA inhibited the expression of TGFβR1 by inhibiting NFY. In addition to TGF-β1 signaling, NFY was involved in fatty acid synthesis and cholesterol synthesis by regulating the transcription factors SREBF1 [22] and SREBF2 (Figure 7F) [23]. We showed that BCAA reduced TGF-β1-induced lipogenesis-related gene expression (such as SCD, SREBF1, and SREBF2) (Figure 5B).

p300 increases WNT/β-catenin signaling by acetylating β-catenin and regulating the interaction of β-catenin and TCF4 (Figure 7F) [24]. Moreover, p300 is involved in gluconeogenesis by cooperating with FoxO1 and PGC1α signaling [25]. A recent report showed that p300 was increased in hepatic steatosis and the p300-C/EBPα/β pathway was activated in the liver of patients with NAFLD [26]. Furthermore, the hepatitis C virus, through its 3′ untranslated region, activated IKK-α, which translocated to the nucleus and induced a CBP/p300-mediated transcriptional program involving SREBPs (Figure 7F) [27]. In this study, we showed that BCAA reduced TGF-β1-induced WNT/β-catenin signaling (such as cyclin D1, β-catenin, EpCAM, and Jagged 1) in Huh-7 cells (Figure 5B). Moreover, BCAA inhibited the TGF-β1-induced malignant transformation of WB-F344 rat liver epithelial stem-like cells (Figure 5D) [9]. Recent reports have shown that TGF-β1 promoted the development of HCC by inducing hepatocyte apoptosis and compensatory proliferation during the early phases of tumorigenesis [9, 28, 29]; therefore, TGF-β1 signaling could be a therapeutic intervention in HCC [30].

It is well known that BCAA immediately phosphorylates mTOR and activates mTORC1 signaling [11]; therefore, we investigated the relationship of mTORC1 and TGF-β1 signaling. Interestingly, activation of mTORC1 by the overexpression of Rheb inhibited the expression of NFYA, p300, p-Smad2, TGFβR1, and collagen 1a2 in Lx-2 cells (Figure 7A and 7B). Conversely, inhibition of mTORC1 by the repression of Raptor increased TGF-β1 signaling further in both HSC and hepatocytes (Figure 7C–7E). The results showed that the suppressive effect of BCAA on TGF-β1 signaling was mTORC1-dependent. Consistent with our findings, a recent report showed that Raptor knock out mice were more susceptible to DEN-induced hepatic fibrosis and HCC [31]. Although the TGF-β1-mTORC1 axis plays essential roles for fibrogenesis in HSC [32, 33], we showed the presence of negative feedback regulation from mTORC1 to TGF-β1 signaling in this study (Supplementary Figure 6B). Activating this feedback regulation by BCAA could reduce the pro-fibrosis signaling in both HSC and hepatocytes that was observed *in vitro* and *in vivo* in this study. Importantly, BCAA could be more beneficial for patients with advanced liver fibrosis whose serum albumin levels are decreased due to reduced mTORC1 signaling in hepatocytes. In these patients, reduced mTORC1 signaling increases TGF-β1 signaling, which further reduces mTORC1 signaling (Supplementary Figure 6B), thereby accelerating hepatocellular death and increasing the incidence of HCC. BCAA supplementation would cancel this vicious cycle. Further studies should be performed to reveal the molecular network between mTOR and TGF-β1 signaling that would be beneficial for the development of novel anti-fibrosis and anti-HCC molecular target drugs.

## MATERIALS AND METHODS

### Animal studies

Ath+HF diet mice were generated as described previously [2]. Male C57BL/6J mice were maintained in a pathogen-free animal facility under a standard 12-h/12-h light/dark cycle. After weaning at week 8, male mice were divided randomly into 3 groups and each group was given one of the following diets for 30 or 60 weeks: (i) basal diet, (ii) Ath+HF diet, or (iii) Ath+HF diet supplemented with 3% BCAA. The mice were killed at 38 weeks (38w) to analyze the progression of hepatic fibrosis or at 68 weeks (68w) to analyze the development of hepatic tumors. The detailed contents of the diets are described in the Supplementary Materials and Methods.

The generation and characterization of platelet-derived growth factor C transgenic (*Pdgf-c Tg*) mice have been described previously [8]. After weaning at week 8, male mice were divided randomly into the following 2 groups: (i) *Pdgf-c Tg* or non-transgenic (*WT*) mice fed a basal diet (CRF-1; Charles River Laboratories Japan) with 3% casein, and (ii) *Pdgf-c Tg* or *WT* mice fed CRF-1 supplemented with 3% BCAA. The mice were killed at week 28 to analyze the progression of hepatic fibrosis and the development of hepatic tumors.

All animal experiments were carried out in accordance with the Guidelines for the Care and Use of Laboratory Animals of the Takara-machi Campus of Kanazawa University, Japan.

### Cell culture

Human hepatic stellate cells (HSC) (Lx-2; kindly provided by Dr. Scott Friedman, Mount Sinai School of Medicine, New York, NY) and a human hepatocellular carcinoma cell line (Huh-7 cells) were maintained in Dulbecco's modified Eagle's medium (DMEM; Gibco BRL, Gaithersburg, MD) containing 10% fetal bovine serum, 1% L-glutamine, and 1% penicillin/streptomycin (normal medium).

### Isolation and culture of mouse HSC

HSC were isolated from C57BL/6J mice by pronase-collagenase liver digestion as reported previously [34].

### Knockdown experiments

Lx-2 cells were transfected with control (Stealth RNAi Negative Control Low GC Duplex #2; Invitrogen, Carlsbad, CA) or regulatory-associated protein of mTOR (Raptor) small interfering RNA (siRNA; Thermo Fisher Scientific K.K., Yokohama, Japan) using the Lipofectamine RNAiMAX Reagent (Invitrogen) according to the manufacturer's instructions. After 24 h, the culture medium was replaced with medium containing 10 ng/mL recombinant human TGF-β1 (R&D, Minneapolis, MN). The cells were harvested for analysis after incubation for 24 h.

### Gene expression profiling

Gene expression profiling of mouse liver was performed using a GeneChip Mouse Gene 1.0 ST Array (Affymetrix, Santa Clara, CA) [34]. Liver tissue from mice fed the basal, Ath+HF, or Ath+HF diet containing 3% BCAA for 30 or 60 weeks was obtained. The expression data were deposited in the Gene Expression Omnibus database (NCBI accession no.: GSE57290). Pathway analysis was conducted using MetaCore (Thomson Reuters, New York, NY). Functional ontology enrichment analysis was conducted to compare the Gene Ontology process distribution of the differentially expressed genes.

### Statistical analysis

The results are expressed as the mean ± standard deviation. Significance was tested by one-way analysis of variance with Bonferroni's method, and differences were considered statistically significant at a P < 0.05.

### Histopathology and immunohistochemical staining, quantitative real-time detection PCR, western blotting, immunofluorescence staining, and promoter analysis

Detailed procedures are described in the Supplementary Materials and Methods.

## SUPPLEMENTARY MATERIALS AND METHODS FIGURES AND TABLES



## Abbreviations

Akt: Thymoma Viral Proto Oncogene
ALT: Alanine Aminotransferase
α-SMA: Alpha Smooth Muscle Actin
Ath+HF Diet: Atherogenic and High-Fat Diet
BCAA: Branched-Chain Amino Acids
DEN: Diethylnitrosamine
EpCAM: Epithelial Cell Adhesion Molecule
ERK: Extracellular Signal-Regulated Kinase
HCC: Hepatocellular Carcinoma
HSC: Hepatic Stellate Cells
IF: Immunofluorescence
IHC: Immunohistochemical
JNK: c-Jun N-Terminal Kinase
mTORC1: Mammalian Target of Rapamycin Complex 1
NAFLD: Non-Alcoholic Fatty Liver Disease
NASH: Nonalcoholic Steatohepatitis
NFY: Nuclear Transcription Factor Y
PDGF: Platelet-Derived Growth Factor
PDGFR: Platelet-Derived Growth Factor Receptor
Rheb: Ras Homolog Enriched in Brain
S6K: Ribosomal Protein S6 Kinase
siRNA: Small Interfering RNA
SREBF: Sterol Regulatory Element Binding Factor
Tg: Transgenic
TGF: Transforming Growth Factor
*WT*: Wild-Type.
