# Supplementary material for: Branched-chain amino acids prevent hepatic fibrosis and development of hepatocellular carcinoma in a non-alcoholic steatohepatitis mouse model
Source: Oncotarget. 2017 Feb 13;8(11):18191–205. doi: 10.18632/oncotarget.15304 (PMC5392319; doi:10.18632/oncotarget.15304)
Supplement: Supplementary file 1 [file oncotarget-08-18191-s001.pdf]

Branched-chain amino acids prevent hepatic fibrosis and development of hepatocellular carcinoma in a non-alcoholic steatohepatitis mouse model

SUPPLEMENTARY DATA

SUPPLEMENTARY MATERIALS AND METHODS

Animal studies

Atherogenic and high-fat (Ath+HF) diet model: male C57BL/6J mice were maintained in a pathogen-free animal facility under a standard 12-h/12-h light/dark cycle. After weaning at week 8, male mice were divided randomly into 3 groups, and each group was fed one of the following diets for 30 or 60 weeks: (i) basal, (ii) Ath+HF, or (iii) Ath+HF supplemented with 3% branched-chain amino acids (BCAA). The basal diet contained 4.3% fat (cocoa butter, soybean oil), 19.2% protein (casein, L-cysteine), 66.4% carbohydrate (corn starch, maltodextrin, sucrose), 4.3% mineral mixture, 0.9% vitamin mixture, 0.2% choline bitartrate, and 4.7% cellulose (Research Diets, New Brunswick, NJ). The Ath+HF diet contained 34.3% fat (cocoa butter, soybean oil), 25.8% protein (25.4% casein, 0.4% L-cysteine), 24.6% carbohydrate (corn starch, maltodextrin, sucrose), 1.3% cholesterol, 0.5% cholic acid, 5.7% mineral mixture, 1.3% vitamin mixture, 0.2% choline bitartrate, and 6.3% cellulose (Research Diets). The Ath+HF diet supplemented with 3% BCAA contained 34.3% fat (cocoa butter, soybean oil), 25.8% protein (3% BCAA, 22.4% casein, 0.4% L-cysteine), 24.6% carbohydrate (corn starch, maltodextrin, sucrose), 1.3% cholesterol, 0.5% cholic acid, 5.7% mineral mixture, 1.3% vitamin mixture, 0.2% choline bitartrate, and 6.3% cellulose (Research Diets). The mice were killed at week 38 to analyze the progression of hepatic fibrosis (N = 16 for each of the basal and Ath+HF diet groups; N = 18 for the BCAA group) or at week 68 to analyze the development of hepatic tumors.

Pdgf-c Tg model

The generation and characterization of *Pdgf-c Tg* mice have been described previously [1]. Non-transgenic WT and *Pdgf-c Tg* mice on a C57BL/6J background were maintained in a pathogen-free animal facility under a standard 12-h/12-h light/dark cycle. After weaning at week 8, male mice were divided randomly into the following 2 groups: (i) *Pdgf-c Tg* or *WT* mice fed a basal diet (CRF-1; Charles River Laboratories Japan K.K., Yokohama, Japan) with 3% casein, and (ii) *Pdgf-c Tg* or *WT* mice fed CRF-1 supplemented with 3% BCAA. The mice were killed at week 28 to analyze the progression of hepatic fibrosis and development of hepatic tumors.

All animal experiments were carried out in accordance with the Guidelines for the Care and Use of Laboratory Animals of the Takara-machi Campus of Kanazawa University, Japan.

Cell culture

Human hepatic stellate cells (Lx-2; kindly provided by Dr. Scott Friedman, Mount Sinai School of Medicine, New York, NY) and Huh-7 cells were maintained in Dulbecco's modified Eagle's medium (DMEM; Gibco BRL, Gaithersburg, MD) containing 10% fetal bovine serum, 1% L-glutamine, and 1% penicillin/streptomycin (normal medium). Amino acid-free medium (ZERO medium) was prepared by mixing 5.81 g nutrition-free DMEM (Nacalai Tesque, Kyoto, Japan), 1.85 g $NaHCO_3$, 1 g glucose, and 0.5 mL of 1 M (mol/L) sodium pyruvate in 500 mL Milli-Q water, then sterilizing with a 0.22-µm filter (Millipore, Billerica, MA). Low amino acid medium (×1/5 DMEM) was prepared by diluting ×1 DMEM with ZERO medium. Powdered BCAA (leucine-isoleucine-valine, 2:1:1.2) (Ajinomoto Pharma, Tokyo, Japan) were freshly dissolved in distilled water at 100 mmol/L, and then applied to the culture medium at 4, 8, or 16 mmol/L. A total of 5.0×10^4 Lx-2 cells were seeded in normal medium at 24 h before performing the experiments. The medium was changed to low amino acid medium and maintained for up to 24 h, and then the medium was replaced with low amino acid medium containing 3 ng/mL recombinant human transforming growth factor (TGF)-β1 (R&D, Minneapolis, MN) with or without BCAA. After incubation for 24 h, the cells were harvested for analysis.

Isolation and culture of mouse hepatic stellate cells

Hepatic stellate cells (HSC) were isolated from C57BL/6J mice. Pronase-collagenase liver digestion was used to isolate HSC from mice. All experiments were replicated at least twice. Freshly isolated HSC suspended in culture medium were seeded in uncoated 24-well plates and incubated at 37°C in a humidified atmosphere of 5% CO_2 for 72 h in normal medium. Non-adherent cells were removed with a pipette and the culture medium was replaced with medium containing 10 ng/mL recombinant

mouse TGF-β1 (R&D) with or without BCAA. The cells were harvested for analysis after incubation for 24 h. Cell viability was assessed by MTT (3-[4,5-dimethylthiazol-2-yl]-2,5-diphenyl tetrazolium bromide) assay after 24 h stimulation by TGF-β1 with or without BCAA.

Histopathology and immunohistochemical staining

Mouse liver tissues were fixed in 10% formalin and stained with hematoxylin and eosin. Liver neoplasms (HCC and liver cell adenoma) were diagnosed according to previously described criteria [2, 3]. Hepatic fibrosis was evaluated by Azan staining. The percent of fibrous areas was calculated microscopically using an image analysis system (BIOREVO BZ-9000; KEYENCE, Osaka, Japan). Immunohistochemical staining was conducted by an immunoperoxidase technique with an Envision Kit (DAKO, Tokyo, Japan). The following primary antibodies were used: rabbit polyclonal PDGFRβ (1:100 dilution; Cell Signaling Technology, Danvers, MA), desmin (1:100 dilution; Cell Signaling Technology), collagen 1 (1:100 dilution; Abcam, Cambridge, MA), and smooth muscle actin (1:100 dilution; Santa Cruz Biotechnology, Dallas, TX).

Quantitative real-time detection PCR

Total RNA was isolated from frozen liver tissue samples using a High Pure RNA Tissue Kit (Roche Applied Science, Indianapolis, IN) according to the manufacturer's protocol. cDNA was synthesized from 100 ng total RNA using a High-capacity cDNA Reverse Transcription Kit (Applied Biosystems, Carlsbad, CA) and then mixed with the TaqMan Universal Master Mix (Applied Biosystems) and each TaqMan probe. The following TaqMan probes were used: PDGFRβ, PDGFB, PDGFC, TGF-β1, α-SMA, collagen 1, collagen 4, Raptor, Timp2, TGFβR1, TGFβR2, SCD, SREBF1, SREBF2, cyclin D1, β-catenin, EpCAM, Jagged1, CD90, and Myc (Applied Biosystems). Relative expression levels were calculated after normalization to glyceraldehyde-3-phosphate dehydrogenase (GAPDH).

Western blotting

Whole-cell lysates from mouse liver were prepared and lysed using the CellLytic MT Cell Lysis Reagent (Sigma-Aldrich Japan K.K., Tokyo, Japan) containing Complete Mini EDTA-free Protease Inhibitor Cocktail Tablets (Roche Applied Science). Cytoplasmic and nuclear protein extracts were prepared using an NE-PER Nuclear Extraction Reagent Kit (Pierce Biotechnology, Rockford, IL). Lx-2 cells were washed in phosphate-buffered saline (PBS) and lysed in RIPA buffer containing Complete Protease Inhibitor Cocktail and PhosSTOP (Roche Applied Science). The membranes were blocked in Blocking One-P (Nacalai Tesque). The following primary antibodies were used: mTOR (1:1,000 dilution), p-mTOR (1:1,000 dilution), p70S6K (1:1,000 dilution), p-p70S6K (1:1,000 dilution), PDGFRβ (1:1,000 dilution), p44/42 MAPK (1:1,000 dilution), p-p44/42 MAPK (1:1,000 dilution), Raptor (1:1,000 dilution), p-Smad2 (1:1,000 dilution), p-Smad3 (1:1,000 dilution), AKT (1:1,000 dilution), p-AKT (1:1,000 dilution), caspase 3 (1:1,000 dilution), lamin A/C (1:1,000 dilution; all Cell Signaling Technology); α-SMA (1:200 dilution; DAKO); p-Smad3L (1:25 dilution; Immuno-Biological Laboratories Co); TGFβR1 (1:250 dilution), TGFβR2 (1:50:)0 dilution), Rheb (1:1000 dilution; all Abcam); NFYA (1:200 dilution), NFYB (1:200 dilution), p300 (1:200 dilution), and GAPDH (1:1,000 dilution; all Santa Cruz).

Immunofluorescence staining

For immunofluorescence staining, the cells were fixed with methanol and then permeabilized with 0.1% Triton-X 100 in PBS. The primary anti-collagen 1 antibody (1:100 dilution; Abcam) was used at a final concentration of 2 μg/mL in PBS containing 2% fetal bovine serum at 4°C for 16 h. Incubation with the Alexa Fluor 488-conjugated secondary antibody (Invitrogen, Carlsbad, CA) at a 500-fold dilution in PBS containing 2% fetal bovine serum antibody was performed for 4 h, and the cells were stained with Hoechst 33258 to visualize nuclear DNA (Vector Laboratories, Burlingame, CA).

Knockdown experiments

Lx-2 or Huh-7 cells were transfected with control (Stealth RNAi Negative Control Low GC Duplex #2; Invitrogen, Carlsbad, CA) or target (Raptor and NFYA) small interfering RNA (Thermo Fisher Scientific K.K., Yokohama, Japan) using the Lipofectamine RNAiMAX Reagent (Invitrogen) according to the manufacturer's instructions. After 24 h, the culture medium was replaced with medium containing 3 ng/mL recombinant human TGF-β1 (R&D) with or without BCAA. The cells were harvested for analysis after incubation for 24 h.

Transfection

Lx-2 cells were transfected with negative control or plasmids encoding the constitutively active form of Ras homolog enriched in brain (Rheb) protein [4] using Lipofectamine 2000 (Invitrogen). After 24 h, the culture medium was replaced with medium containing 3 ng/mL recombinant human TGF-β1 (R&D). The cells were harvested for analysis after incubation for 24 h.

Generating recombinant lentivirus expressing TGF-β1

A cDNA fragment encoding TGF-β1 was generated by PCR from mouse liver tissue-derived cDNA using the forward primer 5′-GGTG GTATACTGAGACACCTTGGTG-3′ and reverse primer 5′-TGTATTTAAGGACACCTGCACCCCC-3′. The TGF-β1 fragment was inserted into the pLVSIN-CMV Pur Vector and cloned in DH5α competent cells. pLVSIN-CMV-TGF-β1 vector or control empty vector, and the three packaging plasmids were co-transfected into Lenti-X 293T cells in 10-cm dishes to generate the recombinant lentiviruses LVSIN-TGF-β1 and LVSIN-cont, respectively. After 48 h, the virus-containing medium was harvested and kept at -80°C until used. TGF-β1 expression levels were evaluated by western-blotting.

Spheroid assay

The rat liver progenitor cell-like cell line WB-F344 (purchased from Japanese Collection of Research Bioresources Cell Bank of the National Institutes of Biomedical Innovation, Health, and Nutrition) was cultured in DMEM (Invitrogen) containing 10% fetal bovine serum (Gibco, Invitrogen). WB-F344 cells were pre-seeded in a 6-well plate, cultured overnight, and then infected with 2 mL lentivirus for 8 h followed by the addition of 2 mL DMEM/F12 medium. After 48 h, the infected cells were plated in 6-well ultra-low attachment culture dishes at 1.0×10^6 cell per well with fresh DMEM containing 5 μg/mL puromycin with or without BCAA (16 mmol/L). After 3 weeks, the number of spheroids was counted.

Gene expression profiling

Gene expression profiling of mouse liver was performed using a GeneChip Mouse Gene 1.0 ST Array (Affymetrix, Santa Clara, CA). Liver tissue from mice fed the basal, Ath+HF, or Ath+HF diet containing 3% BCAA for 30 or 60 weeks was obtained. The expression data were deposited in the Gene Expression Omnibus database (NCBI accession no.: GSE57290). Pathway analysis was conducted using MetaCore (Thomson Reuters, New York, NY). Functional ontology enrichment analysis was conducted to compare the Gene Ontology process distribution of the differentially expressed genes.

Promoter assay

DNA fragments from -1000 to +56 bp relative to the transcription initiation site of TGFβR1 were inserted into pGL4-Basic (Promega Corporation, Madison, WI) using *Kpn*I and *Xho*I sites. Point mutations in the seed region of predicted NFY binding sites were generated using Multisite-QuikChange (Agilent Technologies, Inc., Santa Clara, CA) according to the manufacturer's protocol. All constructs were confirmed by sequencing. Lx-2 cells were cultured for 24 h in 24-well plates, and 200 ng reporter plasmids were co-transfected with 2 ng Renilla luciferase expression vector (pSV40-Renilla) using Lipofectamine 2000 (Invitrogen). The medium was changed to low amino acid medium and maintained for up to 24 h, and the medium was replaced with medium with or without BCAA. After incubation for 24 h, the cells were harvested for analysis.

Statistical analysis

The results are expressed as the mean ± standard deviation. Significance was tested by one-way analysis of variance with Bonferroni's method, and differences were considered statistically significant at a $P < 0.05$.

REFERENCES

1. Campbell JS, Hughes SD, Gilbertson DG, Palmer TE, Holdren MS, Haran AC, Odell MM, Bauer RL, Ren HP, Haugen HS, Yeh MM, Fausto N. Platelet-derived growth factor C induces liver fibrosis, steatosis, and hepatocellular carcinoma. Proceedings of the National Academy of Sciences of the United States of America. 2005; 102:3389-3394.
2. Frith CH, Ward JM, Turusov VS. Tumours of the liver. IARC Sci Publ 1994; 111:223-269.
3. Tholen B, Maronpot RR, Harada T, Nyska A, Rousseaux C, Nolte T, Malarkey DE, Kaufmann W, Kuttler K, Deschl U, Nakae D, Gregson R, Vinlove MP, Brix AE, Singh B, Belpoggi F, et al. Proliferative and nonproliferative lesions of the rat and mouse hepatobiliary system. Toxicol Pathol. 2010; 38:5S-81S.
4. Nishitani S, Horie M, Ishizaki S, Yano H. Branched chain amino acid suppresses hepatocellular cancer stem cells through the activation of mammalian target of rapamycin. PloS one. 2013; 8:e82346.

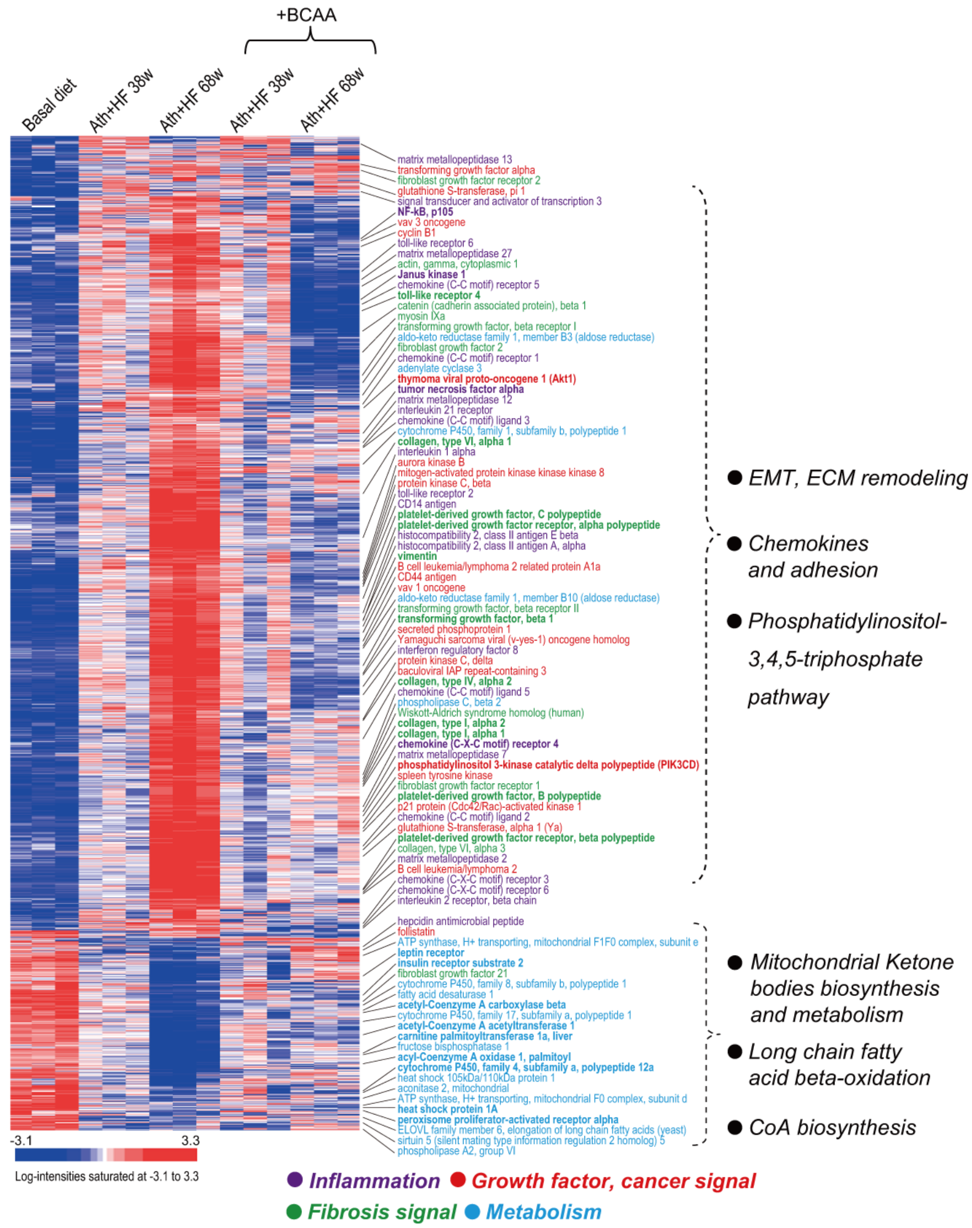

Supplementary Figure 1: One-way hierarchical analysis of 1409 genes expressed differentially between the livers of mice fed the basal diet or the Ath+HF diet at 68w (fold > 1.5, P < 0.005).

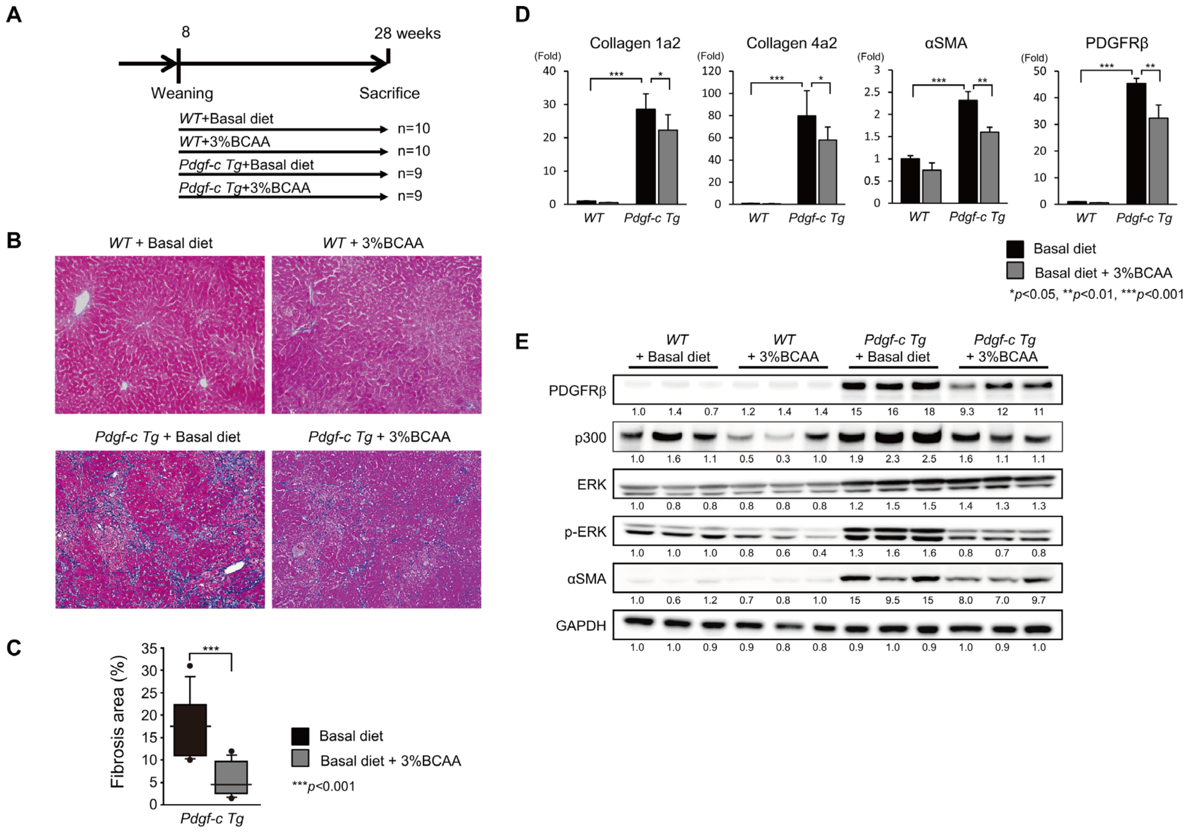

Supplementary Figure 2: Effects of BCAA supplementation on liver fibrosis in *Pdgf-c Tg* and *WT* mice. A. Feeding schedule of the *Pdgf-c Tg* and *WT* mice. After weaning, male mice were divided randomly into 2 groups: (i) *Pdgf-c Tg* or *WT* mice that were fed a basal diet (including 3% casein) and (ii) *Pdgf-c Tg* or *WT* mice fed a 3% BCAA-containing diet. **B.** Azan staining of *Pdgf-c Tg* and *WT* mice livers fed different diets at 28w. **C.** Densitometric analysis of *Pdgf-c Tg* mice liver fibrotic areas at 28w. **D.** Relative expression of mRNA for collagen 1a2, collagen 4a2, α-SMA, and PDGFRβ in livers of *Pdgf-c Tg* and *WT* mice fed the basal or 3% BCAA-containing diet (N = 4). **E.** Western blotting of PDGFRβ, p300, p-ERK, and α-SMA in livers of *Pdgf-c Tg* and *WT* mice fed the basal or 3% BCAA-containing diet.

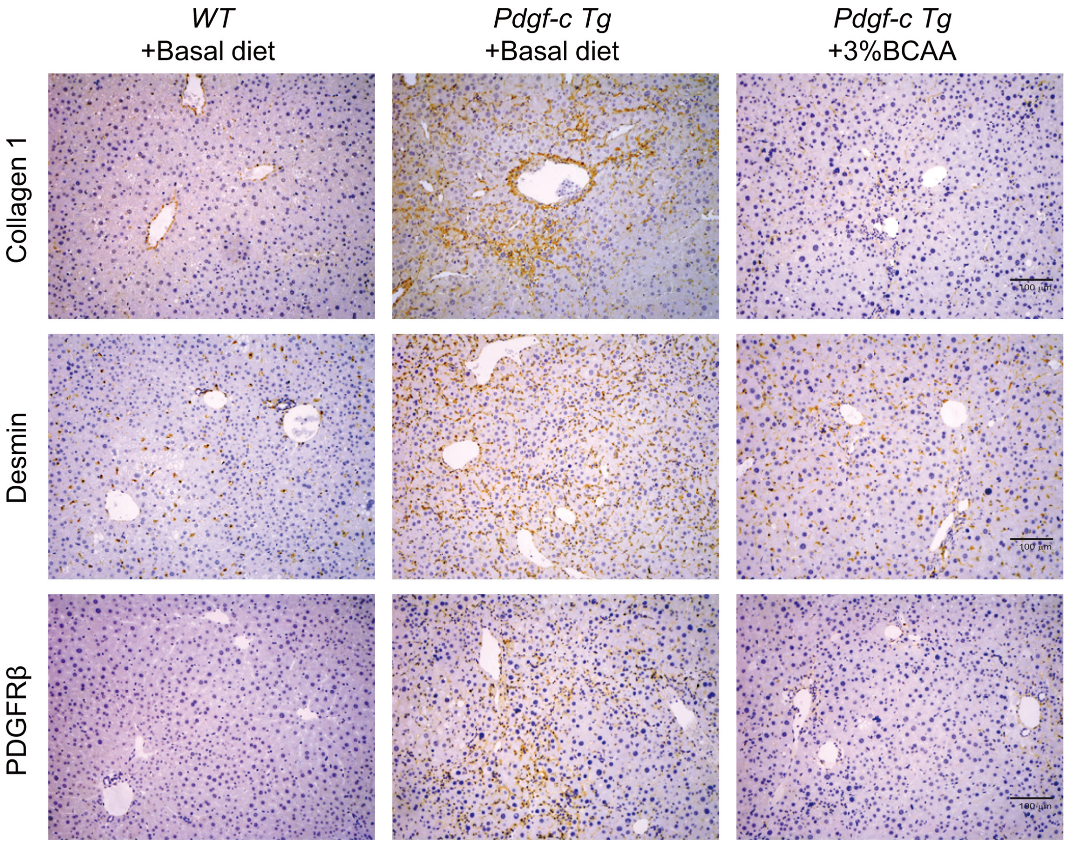

Supplementary Figure 3: IHC staining for collagen 1, desmin, and PDGFRβ expression in the livers of *Pdgf-c Tg* and *WT* mice fed with the basal or 3% BCAA-containing diet.

A

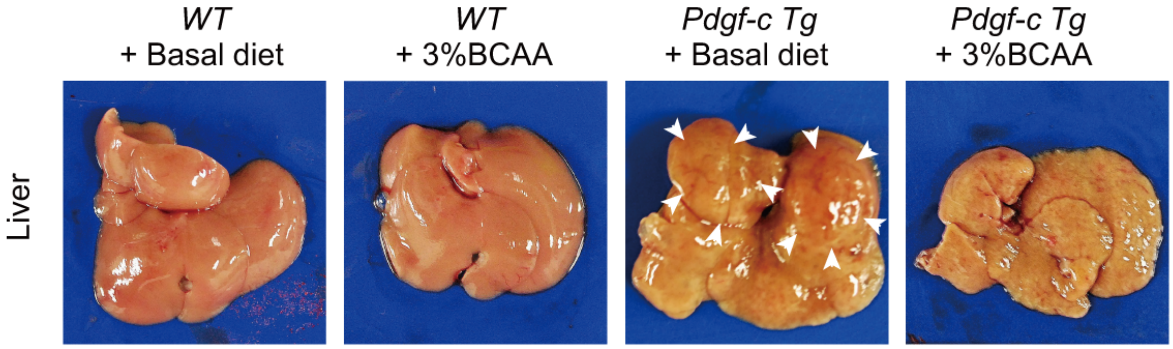

B

	No.	Tumor Adenoma	Tumor HCC	Total incidence (%)
WT + Basal diet	10	0	0	0/10(0)
WT + 3%BCAA	10	0	0	0/10(1)
Pdgf-c Tg + Basal diet	9	8	1	9/9(100) ┐ ***
Pdgf-c Tg + 3%BCAA	9	1	0	1/9(11.1) ┘

***p<0.001

C

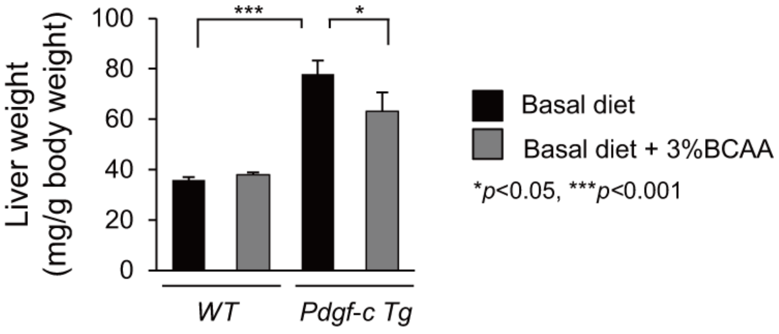

Supplementary Figure 4: Effects of BCAA supplementation on liver tumorigenesis in *Pdgf-c Tg* and *WT* mice. A. Macroscopic findings of livers. **B.** Incidence of hepatic tumors (adenoma or HCC) in livers of *Pdgf-c Tg* and *WT* mice fed the basal or 3% BCAA-containing diet. **C.** Liver weights of *Pdgf-c Tg* and *WT* mice fed the basal or 3% BCAA-containing diet.

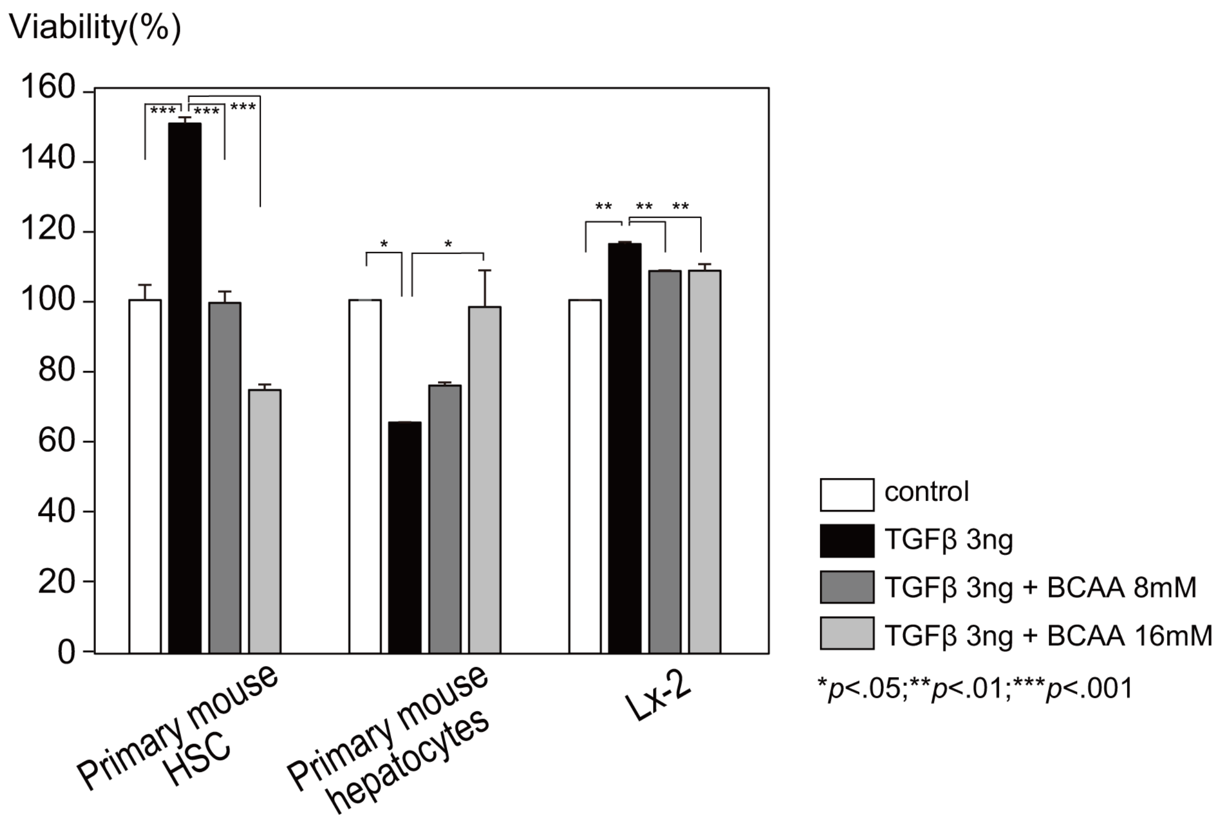

Supplementary Figure 5: Cell viability of primary mouse HSC, primary mouse hepatocytes, and Lx-2 cells treated with TGF-β1 in the presence or absence of BCAA. The cells were treated with TGF-β1 (3 ng/mL) and different concentrations of BCAA (0, 8, and 16 mM). At 24 h after incubation, cell viability was assessed by MTT assay.

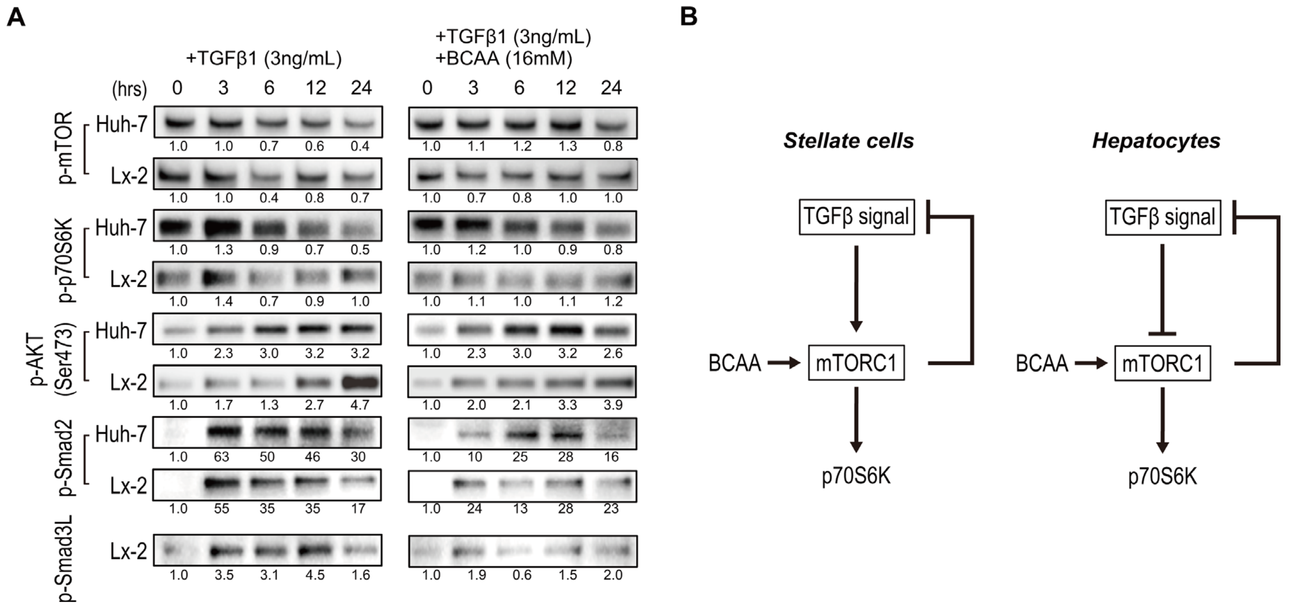

Supplementary Figure 6: Differential mTORC1 signaling induced by TGF-β1 in HSC and hepatocytes. A. Time course of mTORC1 signaling induced by TGF-β1 in the presence or absence of BCAA was evaluated in Lx-2 and Huh-7 cells. **B.** Differential regulation between TGF-β1 and mTORC1 signaling in HSC and hepatocytes.

Supplementary Table 1: Laboratory data in serum of 12 weeks and 68 weeks old mice fed with basal diet, Ath+HF diet and Ath+HF supplemented with BCAA

	12weeks			68weeks		
	**Basal diet**	**Ath & HF**	**Ath & HF + BCAA**	**Basal diet**	**Ath & HF**	**Ath & HF + BCAA**
Serum ALT (U/L)	93.5 ± 64.1	210.9 ± 98.1^{+}	176.9 ± 69.6	59.3 ± 21.2	431.7 ± 178.4***	93.1 ± 26.4¶¶¶
Plasma triglycerides (mg/dL)	17.7 ± 5.4	5.3 ± 1.9^{+++}	5.4 ± 3.0^{+++}	26.9 ± 7.6	14.9 ± 5.8	14.4 ± 10.2
Plasma Total Cholesterol (mg/dL)	125.2 ± 18.2	217.1 ± 18.3^{+++}	195.5± 21.4^{+++}, §	112.6 ± 28.5	239.0 ± 22.4***	204.6 ± 51.6***, ¶
Plasma Free Cholesterol (mg/dL)	29.7 ± 5.6	48.4 ± 5.5^{+++}	43.6 ± 5.7^{+++}	16.6 ± 54.1	54.1 ± 10.3***	39.8 ± 9.5***, ¶¶¶
Plasma FFA (mEq/L)	1.1 ± 0.3	0.7 ± 0.1^{++}	0.8 ± 0.2^{+}	1.8 ± 0.2	1.1 ± 0.2***	0.78 ± 0.2***, ¶
Plasma insulin (µU/mL)	N.D.	N.D.	N.D.	33.3 ± 16.7	21.9 ± 15.1	37.3 ± 19.4
Fasting blood glucose (mg/dL)	N.D.	N.D.	N.D.	154.1 ± 29.8	163.3 ± 37.9	181.4 ± 71.4
HOMA-IR	N.D.	N.D.	N.D.	12.6 ± 6.6	9.5 ± 7.4	18.1 ± 12.8

Data are mean ± SD.
Significantly different from Basal diet value: ^{+}p<.05;^{++}p<.01;^{+++}p<.001 in 12 weeks old mice.
Significantly different from Ath+HF value: §*p*<.05 in 12 weeks old mice.
Significantly different from Basal diet value: **p*<.05;***p*<.01;****p*<.001 in 68 weeks old mice.
Significantly different from Ath+HF value: ¶*p*<.05;¶¶*p*<.01;¶¶¶*p*<.001 in 68 weeks old mice.
Abbreviations: FFA, free fatty acid; HOMA-IR, homeostasis model assessment insulin resistance; N.D., Not determined.

Supplementary Table 2: Pathway analysis of differentially expressed genes in the liver of Ath+HF diet and BCAA administration

Pathway Name	p-values	Gene Symbole (Affy ST array ID ver 1.0)			
**Up-regulated pathway map in Ath+HF diet and repressed by BCAA administration**					
1. Cell adhesion_ECM remodeling	1.39E-03	COL1A1(10380419)	COL1A2(10536220)	COL4A2(10570068)	MMP13(10583044)
		CD44(10485405)	KLK1(10369541)	NID1(10403584)	
2. Cell adhesion_Chemokines and adhesion	5.18E-03	CCL2(10379511)	CCR1(10598004)	NFKB1(10502299)	CTNNB1(10590325)
		PAK1(10555118)	LIMK1(10383684)		
3. Development_Regulation of EMT	1.69E-02	EGFR1(10570982)	TGFB1(10551185)	TGFBR1(10504817)	VIM(10469322)
		PDGFB(10430660)	PDGFRB(10456046)	FGF2(10491699)	
**Up-regulated metabolic network in Ath+HF diet and repressed by BCAA administration**					
1. Phosphatidylinositol-4,5-diphosphate pathway	1.82E-18	PIK3CG(10399924)	PIK3CD(10518686)	PLCG2(10575799)	PLEK(10384458)
2. Sphingomyelin pathway	2.52E-11	SMPD3(10581479)	PSAP(10363430)	LRP1(10373223)	PLAUR(10550906)
3. Phosphatidylinositol-3,4,5-triphosphate pathway	8.92E-10	AKT1(10402766)	AKT3(10360506)	BTK(10606694)	VAV1(10446253)
**Down-regulated pathway map in Ath+HF diet and rescued by BCAA administration**					
1. Mitochondrial ketone bodies biosynthesis and metabolism	2.39E-04	ACAT1(10593591)	HADHA(10528929)	HADHB(10520467)	HMGCL(10412466)
		BDH1(10434934)	HMGCS2(10494643)		
2. Mitochondrial long chain fatty acid beta-oxidation	1.99E-03	CPT1A(10460157)	ACSL1(10571657)	ACSL5(10464045)	HADH(10502205)
3. CoA biosynthesis	1.80E-02	ACOT1(10397148)	ACOT8(10489553)	ACOT2(10397145)	VNN1(10362138)
		PANK1(10467162)			
**Down-regulated metabolic network in Ath+HF diet and rescued by BCAA administration**					
1. Lipid metabolism_Fatty Acid Beta-oxydation	1.91E-12	ACOX1(10393177)	ACADL(10355246)	ACADS(10532926)	ACAA1(10590137)
		HSD17B10(10602592)			
2. Lipid metabolism_n-6 Poliunsaturated fatty acid biosynthesis	4.56E-06	ELOVL2(10408838)	ELOVL5(10587284)	FADS1(10461439)	FADS2(10465895)

Supplementary Table 3: Laboratory data in serum of 28 weeks old *WT* and *PDGF-c* Tg mice fed with basal diet and basal diet supplemented with BCAA

	WT		*PDGF-c Tg*	
	**Basal diet**	**3% BCAA**	**Basal diet**	**3% BCAA**
Serum ALT (U/L)	52.4 ± 39.7	67.3 ± 30.2	222.4 ± 65.8***	225.3 ± 80.4***
Plasma triglycerides (mg/dL)	30.8 ± 10.4	27.7 ± 5.1	47.7 ± 11.8**	41.3 ± 16.87
Plasma Total Cholesterol (mg/dL)	88.1 ± 11.5	88.0 ± 12.7	186.3 ± 38.3***	129.6 ± 66.8¶
Plasma Free Cholesterol (mg/dL)	18.8 ± 2.8	18.9 ± 1.6	72.7 ± 22.8***	53.4 ± 23.6¶
Plasma FFA (mEq/L)	1.5 ± 0.2	1.5 ± 0.2	1.7 ± 0.2*	1.5 ± 0.1¶
Fasting blood glucose (mg/dL)	80.9 ± 26.4	81.7 ± 24.7	83.9 ± 22.2	81.0 ± 22.4

Data are mean ± SD.
Significantly different from Basal diet value in *WT* mice: *$p<.05$;**$p<.01$;***$p<.001$ in *PDGF-c* Tg mice.
Significantly different from Basal diet value in PDGF-c Tg: ¶$p<.05$ in *PDGF-c* Tg mice.
Abbreviations: FFA, free fatty acid.
